# Evaluation of five different renal recovery definitions for estimation of long-term outcomes of cardiac surgery associated acute kidney injury

**DOI:** 10.1186/s12882-019-1613-6

**Published:** 2019-11-21

**Authors:** Jiarui Xu, Xialian Xu, Bo Shen, Yamin Zhuang, Lan Liu, Yimei Wang, Yi Fang, Zhe Luo, Jie Teng, Chunsheng Wang, Claudio Ronco, Jiawei Yu, Xiaoqiang Ding

**Affiliations:** 1 Department of Nephrology, Zhongshan Hospital, Shanghai Medical College, Fudan University, No. 180 Fenglin Road, Shanghai, 200032 China; 2 Shanghai Medical Center of Kidney, No. 180 Fenglin Road Shanghai, Shanghai, 200032 China; 3 Shanghai Institute for Kidney and Dialysis, No. 180 Fenglin Road Shanghai, Shanghai, 200032 China; 4 Shanghai Key Laboratory of Kidney and Blood Purification, No. 180 Fenglin Road Shanghai, Shanghai, 200032 China; 5 Department of Critical Care Medicine, Zhongshan Hospital, Shanghai Medical College, Fudan University, No. 180 Fenglin Road, Shanghai, 200032 China; 6 Department of Nephrology, Xiamen Branch, Zhongshan Hospital, Fudan University, No. 668 Jinhu Road, Xiamen, 361015 Fujian China; 7 Department of Cardiovascular Surgery, Zhongshan Hospital, Shanghai Medical College, Fudan University, No. 180 Fenglin Road, Shanghai, 200032 China; 8 Department of Nephrology, Dialysis and Transplantation San Bortolo Hospital, Via Rodolfi, 37, 36100 Vicenza, Italy; 9 International Renal Research Institute of Vicenza (IRRIV), |Via Rodolfi, 37, 36100 Vicenza, Italy

**Keywords:** Acute kidney injury, Cardiac surgery, Long-term outcome, Renal recovery

## Abstract

**Background:**

The commonly used recommended criteria for renal recovery are not unequivocal. This study compared five different definitions of renal recovery in order to evaluate long-term outcomes of cardiac surgery associated acute kidney injury (CSA-AKI).

**Methods:**

Patients who underwent cardiac surgery between April 2009 and April 2013 were enrolled and divided into acute kidney injury (AKI) and non-AKI groups. The primary endpoint was 3-year major adverse events (MAEs) including death, new dialysis and progressive chronic kidney disease (CKD). We compared five criteria for complete renal recovery: Acute Renal Failure Trial Network (ATN): serum creatinine (SCr) at discharge returned to within baseline SCr + 0.5 mg/dL; Acute Dialysis Quality Initiative (ADQI): returned to within 50% above baseline SCr; Pannu: returned to within 25% above baseline SCr; Kidney Disease: Improving Global Outcomes (KDIGO): eGFR at discharge ≥60 mL/min/1.73 m^2^; Bucaloiu: returned to ≥90% baseline estimated glomerular filtration rate (eGFR). Multivariate regression analysis was used to compare risk factors for 3-year MAEs.

**Results:**

The rate of complete recovery for ATN, ADQI, Pannu, KDIGO and Bucaloiu were 84.60% (*n* = 1242), 82.49% (*n* = 1211), 60.49% (*n* = 888), 68.60% (*n* = 1007) and 46.32% (*n* = 680). After adjusting for confounding factors, AKI with complete renal recovery was a risk factor for 3-year MAEs (OR: 1.69, 95% CI: 1.20–2.38, *P* <  0.05; OR: 1.45, 95% CI: 1.03–2.04, *P* <  0.05) according to ATN and ADQI criteria, but not for KDIGO, Pannu and Bucaloiu criteria. We found that relative to patients who recovered to within 0% baseline SCr or recovered to ≥100% baseline eGFR, the threshold values at which significant differences in 3-year MAEs were observed were > 30% or > 0.4 mg/dL above baseline SCr or < 70% of baseline eGFR.

**Conclusions:**

ADQI or ATN-equivalent criteria may overestimate the extent of renal recovery, while KDIGO, Pannu and Bucaloiu equivalent criteria may be more appropriate for clinical use. Our analyses revealed that SCr at discharge > 30% or > 0.4 mg/dL of baseline, or eGFR < 70% of baseline led to significant 3-year MAE incidence differences, which may serve as hints for new definitions of renal recovery.

## Background

During the last few decades, many studies have shown that patients who survive acute kidney injury (AKI) have a greater risk of developing chronic kidney disease (CKD), end stage renal disease (ESRD) and other adverse outcomes compared to patients without AKI [1].

Regarding AKI after cardiac surgery, efforts have often been made to develop predictive models [2], finding long term factors for mortality and progressive CKD [3], as well as evaluating goal-directed renal replacement therapy (GDRRT) in order to improve AKI therapy and to prevent postoperative complications [4, 5]. However, the definition of renal recovery has not been unified yet [6–10]. Compared with studies about the definition and validation of AKI, there are few studies on renal recovery from AKI and published reports have mainly focused on renal recovery after renal replacement therapy (RRT) [11]. Studies have shown that early nephrology follow-up after hospitalization for AKI and early referral to a nephrologist is associated with improved survival [12, 13]. Kirwan et al. studied patients, who survived AKI and required RRT in an intensive care unit (ICU) in East London and found only 57% had serum creatinine (SCr) levels measured within 3–6 months after discharge and that only 12% received a specialist nephrology follow-up [14]. Lack of knowledge of renal recovery can decrease the awareness of follow-up and intervention in the recovery phase of AKI to CKD. Therefore, a valid definition of renal recovery is needed in order to enhance the interventions. According to Kellum et al., a good definition of renal recovery from AKI should include four main domains: inception (starting point for recovery); magnitude (threshold for recovery); timing (when recovery is assessed), and confounding factors [15]. However, the commonly used recommended criteria for renal recovery such as Acute Dialysis Quality Initiative (ADQI), Kidney Disease: Improving Global Outcomes (KDIGO) and Acute Renal Failure Trial Network (ATN) are not unequivocal. In the present study, the effects of different renal recovery definitions were evaluated for estimation of long-term outcomes of cardiac surgery associated AKI (CSA-AKI).

## Methods

### Patients

The ethical committee of Shanghai Zhongshan Hospital approved the study (No. B2017–039) and written informed consent was obtained from all patients. The study was conducted in accordance with the Declaration of Helsinki regarding the ethical principles for medical research involving human subjects. We collected data from patients who underwent cardiac surgery in Shanghai Zhongshan Hospital between April 2009 and April 2013. Exclusion criteria included: age < 18 years; preoperative CKD; survived < 24 h in ICU. In total, 3869 patients meeting the entry requirements were enrolled in the study.

### Study design

In this single center retrospective observational study, we chose five commonly used definitions of renal recovery to compare their accuracy in evaluating the long-term outcomes of CSA-AKI patients.

### Definitions

The five definitions of “complete renal recovery” were: 1) ATN [10]: SCr at discharge returned to within baseline SCr + 0.5 mg/dL; 2) ADQI [7]: returned to within 50% above baseline SCr; 3) Pannu et al. [9]: returned to within 25% above baseline SCr; 4) KDIGO [6]: estimated glomerular filtration rate (eGFR) at discharge ≥60 mL/min/1.73 m^2^ (Chronic Kidney Disease Epidemiology Collaboration equation (CKD-EPI formula)) and 5) Bucaloiu et al. [8]: eGFR at discharge ≥90% of baseline eGFR. The five definitions of “partial renal recovery” were no RRT but failed to meet the criteria for complete renal recovery.

AKI was defined according to the KDIGO 2012 criteria as the absolute value of the SCr increase ≥26.5 mmol/L within 48 h or an increase > 50% compared to the baseline values within 7 days after surgery, or a urine output < 0.5 mL/kg/h ≥ 6 h [6]. CKD was diagnosed according to the KDIGO criteria [16]. The estimated glomerular filtration rate (eGFR) was calculated using the CKD-EPI formula: eGFR = a × (SCr/b)^c^ × (0.993)^age^ where a = 144 (females)/141 (males), b = 0.7 (females)/0.9 (males), c = female: − 0.329 (SCr ≤ 0.7 mg/dL)/− 1.209 (SCr > 0.7 mg/dL), male: − 0.411 (SCr ≤ 0.7 mg/dL)/− 1.209 (SCr > 0.7 mg/dL). In our urine test, “-” means “no proteinuria”, and 1+ indicates 30 mg/dL (< 0.5 g/day) and 2+ 100 mg/dL (0.5-1 g/day), 3+ 300 mg/dL (1-2 g/day) as well as 4+ more than 1000 mg/dL (more than 2 g/day) and is noted as mild to heavy proteinuria. Proteinuria was defined once 1+ appeared. Progressive CKD was defined as CKD stages 4–5 (GFR ≤ 30 mL/min/1.73 m^2^) including ESRD (received maintenance renal replacement therapy or renal transplantation) [1]. AKI stage 1 without RRT was defined as mild AKI and AKI stage 2–3 was defined as severe AKI.

### Groups

All patients who underwent cardiac surgery were divided into AKI and non-AKI groups. AKI groups were subdivided into mild AKI and severe AKI groups and preoperative eGFR ≥90 mL/min/1.73 m^2^ or preoperative eGFR < 90 mL/min/1.73 m^2^ groups.

### Data collection

We collected basic patient characteristics including age, gender, preoperative comorbidities, cardiac function and renal function. Perioperative data included the type of surgery, cardiopulmonary bypass duration, aortic clamping duration and intraoperative hypotension. Postoperative data included ICU and the length of hospital stay.

### Outcomes

The primary endpoint for long-term outcomes was major adverse events (MAE) including all-cause mortality, new dialysis and progressive CKD [17]. All the surviving patients were followed up at least 3 yrs after surgery by telephone, email or as outpatients.

### Statistical analysis

Statistical analysis was conducted with SPSS Statistics for Windows (Version 17.0. Chicago: SPSS Inc.). Normally distributed data are presented as means ± SD; groups were compared using 2 independent sample *t*-tests or analysis of variance (ANOVA). Nonparametric data are expressed as medians (*P*_25_, *P*_75_). The Wilcoxon test was used to assess two dependent variables, a nonparametric Mann–Whitney test for independent variables, and a chi-squared test for group comparisons. Multivariate Cox regression analyses were performed to investigate the effects of multiple factors on MAEs. Missing cases were deleted directly. A *P*-value < 0.05 was considered to be statistically significant.

## Results

### Baseline characteristics

A total of 3869 patients were divided into AKI (*n* = 1468, 37.94%) and non-AKI groups (*n* = 2401, 62.06%). The cases in AKI stages 1, 2 or 3 were 983 (66.96%), 264 (17.98%) and 221 (15.05%), respectively. Between the AKI and non-AKI groups, there were significant differences in age (57.00 ± 12.00 vs 53.00 ± 14.00 years, *P* <  0.01), male gender (67.57% vs 53.27%, *P* <  0.01) and body mass index (BMI) (23.40 ± 3.70 vs 22.80 ± 3.40 kg/m^2^, *P* <  0.01). Comorbidities including hypertension and chronic heart failure in the AKI group occurred more frequently than in the non-AKI group. Preoperative blood urea nitrogen (BUN), SCr, uric acid and the proportion of proteinuria in the AKI group were significantly higher than in the non-AKI group, whereas eGFR was significantly lower in the AKI group and the proportion of preoperative eGFR ±90 mL/min/1.73 m^2^ was significantly higher in the non-AKI group. The cardiopulmonary bypass and aortic cross-clamping durations in the AKI group were much longer than in the non-AKI group (Table 1).

**Table 1:** Baseline characteristics of patients who underwent cardiac surgery

	All *N* = 3869	Non-AKI *N* = 2401	AKI *N* = 1468
Age (years)	54.00 ± 14.00	53.00 ± 14.00	57.00 ± 12.00^**^
Male (n, %)	2271 (58.70%)	1279 (53.27%)	992 (67.57%)^**^
BMI (kg/m^2^)	23.00 ± 3.50	22.80 ± 3.40	23.40 ± 3.70^**^
Hypertension (n, %)	1135 (29.34%)	649 (27.03%)	486 (33.11%)^**^
Diabetes (n, %)	365 (9.43%)	212 (8.83%)	153 (10.42%)
Preoperative angiography (n, %)	1568 (40.53%)	926 (38.57%)	642 (43.73%)^**^
NYHA III-IV (n, %)	2289 (59.16%)	1352 (56.31%)	937 (63.83%)^**^
Preoperative BUN (mmol/L)	6.20 ± 1.90	6.00 ± 1.80	6.60 ± 2.00^**^
Preoperative SCr (μmol/L)	74.50 ± 15.50	73.20 ± 14.80	76.60 ± 16.30^**^
Preoperative uric acid (μmol/L)	356.60 ± 104.60	343.40 ± 98.20	378.20 ± 111.00^**^
Preoperative eGFR (mL/min/1.73 m^2^)	94.50 ± 22.20	95.10 ± 21.10	93.50 ± 23.80^*^
eGFR ≥90 mL/min/1.73 m^2^ (n, %)	2023 (52.29%)	1303 (54.27%)	720 (49.05%)^**^
Preoperative proteinuria (n, %)	248 (6.41%)	130 (5.41%)	118 (8.04%)^**^
Type of surgery:			
Valve (n, %)	2238 (57.84%)	1353 (56.35%)	885 (60.29%)^*^
CABG (n, %)	734 (18.97%)	499 (20.78%)	235 (16.01%)^**^
Aneurysm (n, %)	199 (5.14%)	85 (3.54%)	114 (7.77%)^**^
Valve + CABG (n, %)	156 (4.03%)	67 (2.79%)	89 (6.06%)^**^
Cardiopulmonary bypass duration (min)	99.00 ± 40.50	91.00 ± 34.00	114.00 ± 45.00^**^
Aortic cross clamping duration (min)	59.20 ± 26.90	55.00 ± 24.00	65.00 ± 29.00^**^

^*^ *P* <  0.05 compared with non-AKI group; ^**^ *P* <  0.001 compared with non-AKI group

*AKI*: Acute kidney injury; *BMI*: Body mass index; *BUN*: Blood urea nitrogen; *CABG*: Coronary artery bypass grafting; *eGFR*: Estimated glomerular filtration rate; *NYHA*: New York heart association; *SCr*: Serum creatinine

### Short- and long-term outcomes

The in-hospital mortality was 2.97% (*n* = 115) and was significantly higher in the AKI than in the non-AKI group (6.70% vs 0.70%, *P* <  0.001). Mortality was increased along with AKI severity (AKI 1, 2, 3: 1.40, 4.90, 32.60%, respectively, *P* <  0.001). The in-hospital RRT incidence was 3.57% (*n* = 138) and was ceased for all RRT patients at hospital discharge. The length of ICU and hospital stays was significantly longer in the AKI group compared to the non-AKI group (70 [42, 119] vs 49 [32, 72] h, *P* <  0.001; 18 ± 13 vs 14 ± 10 d, *P* <  0.001). The 1, 2 and 3-year overall survival rates in the AKI group were significantly lower than in the non-AKI group (85.90% vs 98.10%, *P* <  0.001, 82.30 vs 93.70%, *P* <  0.001, 79.90% vs 90.50%, *P* <  0.001). There were no new dialysis cases in either the AKI or non-AKI groups. The incidence of progress into CKD in the AKI group was significantly higher than in the non-AKI group (0.30% vs 7.70%, *P* <  0.001). The incidence of 3-year MAE in the AKI group was significantly higher than in the non-AKI group (27.8% vs 9.7%, *P* <  0.001) (Table 2).

**Table 2:** Short- and long-term outcomes of AKI vs non-AKI patients

	Non-AKI *N* = 2401	AKI *N* = 1468	*P*-value
*Short-term outcomes*			
In-hospital mortality (n, %)	17 (0.7%)	98 (6.7%)	< 0.001
Length of ICU stay (h)	49 [32, 72]	70 [42, 119]	< 0.001
Length of hospital stay (d)	14 ± 10	18 ± 13	< 0.001
*Long-term outcomes*			
Progressive CKD (n, %)	7 (0.3%)	113 (7.7%)	< 0.001
New dialysis (n, %)	0	0	N/A
1-year overall survival (n, %)	2356 (98.1%)	1261 (85.9%)	< 0.001
2-year overall survival (n, %)	2250 (93.7%)	1208 (82.3%)	< 0.001
3-year overall survival (n, %)	2174 (90.5%)	1173 (79.9%)	< 0.001
3-year MAE (n, %)	234 (9.7%)	408 (27.8%)	< 0.001

*AKI*: Acute kidney injury; *ICU*: Intensive care unit; *CKD*: Chronic kidney disease; *MAE*: Major adverse events;

### Renal recovery

According to the five different definitions of renal recovery, the ATN, ADQI, Pannu, KDIGO and Bucaloiu proportions of complete recovery were 84.60% (*n* = 1242), 82.49% (*n* = 1211), 60.49% (*n* = 888), 68.60% (*n* = 1007), 46.32% (*n* = 680), whereas the proportions of partial renal recovery were 15.40% (*n* = 226), 17.51% (*n* = 257), 39.51% (*n* = 580), 31.40% (*n* = 461) and 53.68% (*n* = 788).

In a sub-group analysis, we found that using five definitions of renal recovery, complete renal recovery mostly derived from mild AKI group, while most patients in the severe AKI group had partial renal recovery **(**Table 3**)**.

**Table 3:** Renal recovery of overall AKI, mild AKI and severe AKI patients under five definitions (n = 1468)

	ATN	ADQI	Pannu et al.	KDIGO	Bucaloiu et al.
Total (n = 1468)					
Complete recovery	1242 (84.60%)	1211 (82.49%)	888 (60.49%)	1007 (68.60%)	680 (46.32%)
Partial recovery	226 (15.40%)	257 (17.51%)	580 (39.51%)	461 (31.40%)	788 (53.68%)
Mild AKI (*n* = 973)					
Complete recovery (n, %)	920 (94.55%)	896 (92.09%)	661 (67.93%)	736 (75.64%)	501 (51.49%)
Partial recovery (n, %)	53 (5.45%)	77 (7.91%)	312 (32.07%)	237 (24.36%)	472 (48.51%)
Severe AKI (*n* = 495)					
Complete recovery (n, %)	322 (65.05%)	315 (63.64%)	227 (45.86%)	271 (54.75%)	179 (36.16%)
Partial recovery (n, %)	173 (34.95%)	180 (36.36%)	268 (54.14%)	224 (45.25%)	316 (63.84%)

*ADQI*: Acute Dialysis Quality Initiative; *AKI*: acute kidney injury; *ATN*: Acute Renal Failure Trial Network; *KDIGO*: Kidney Disease: Improving Global Outcomes

In another sub-group analysis, patients were divided into a preoperative eGFR ≥90 mL/min/1.73 m^2^ and a preoperative eGFR < 90 mL/min/1.73 m^2^ group. Under five different definitions of complete renal recovery, SCr at discharge in the eGFR ≥90 mL/min/1.73 m^2^ groups was significantly lower and eGFR at discharge was significantly higher than in the eGFR < 90 mL/min/1.73 m^2^ groups **(**Table 4**)**.

**Table 4:** Renal function at discharge in complete recovery patients between preoperative eGFR ≥90 mL/min/1.73 m^2^ vs eGFR < 90 mL/min/1.73 m^2^ groups under five definitions

	ATN	ADQI	Pannu	KDIGO	Bucaloiu
SCr at discharge (mg/dL)					
Preoperative eGFR values ≥90 mL/min/1.73 m^2^	0.86 ± 0.23^*^	0.85 ± 0.23^*^	0.77 ± 0.19^*^	0.84 ± 0.21^*^	0.71 ± 0.15^*^
Preoperative eGFR values < 90 mL/min/1.73 m^2^	1.09 ± 0.27	1.11 ± 0.31	1.00 ± 0.27	0.96 ± 0.20	0.93 ± 0.20
eGFR at discharge (mL/min/1.73 m^2^)					
Preoperative eGFR values ≥90 mL/min/1.73 m^2^	95.94 ± 22.75^*^	97.62 ± 21.68^*^	105.50 ± 17.98^*^	98.1 ± 21.05^*^	111.17 ± 14.98^*^
Preoperative eGFR values < 90 mL/min/1.73 m^2^	69.99 ± 18.60	69.46 ± 18.96	76.90 ± 16.55	80.16 ± 14.58	82.78 ± 14.73

^*^ *P* < 0.05 comparing with eGFR < 90 mL/min/1.73 m^2^ groups

*ADQI*: Acute Dialysis Quality Initiative; *ATN*: Acute Renal Failure Trial Network; *eGFR*: Estimated glomerular filtration rate; *KDIGO*: Kidney Disease: Improving Global Outcomes; *SCr*: serum creatinine

### Risk factors for long-term outcomes

**Fig. 1:**
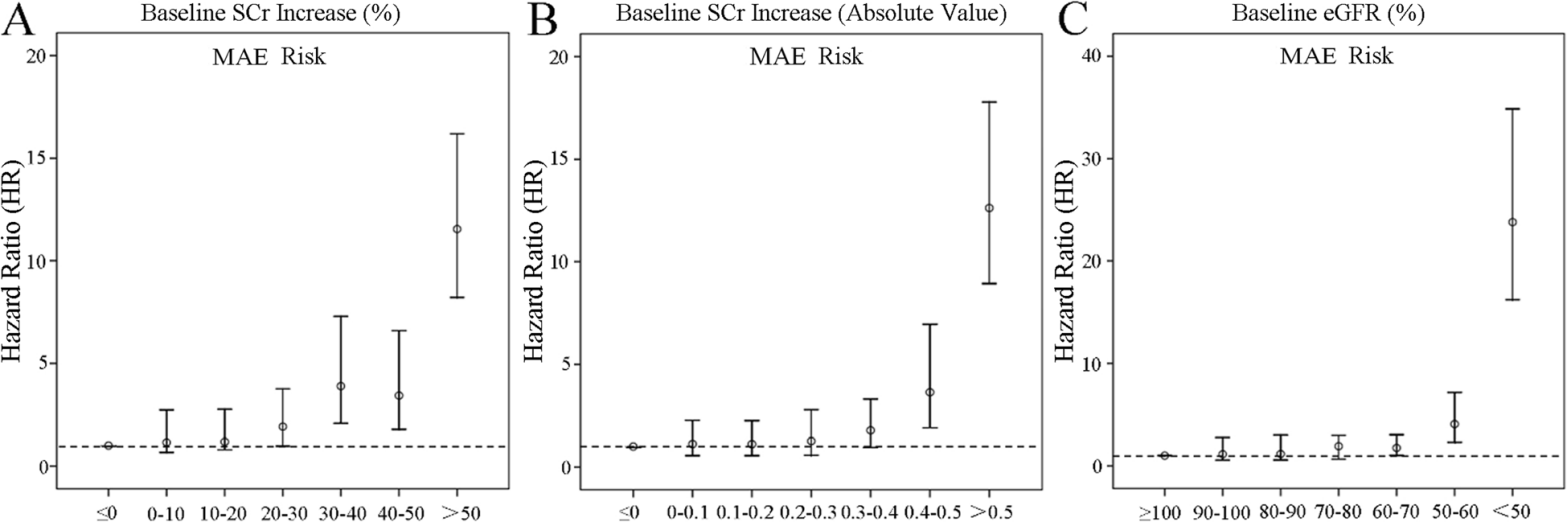
Threshold value for 3-year MAE development. Figure legend: After adjustments for age, BMI, diabetes, NYHA III-IV, RRT, we found that relative to the patients who recovered to within 0% of the baseline SCr, the threshold value at which a significant difference in 3-year MAE was observed was more than 30% (Fig. 1a) or more than 0.4 mg/dL (Fig. 1b) above baseline SCr. Relative to the patients who recovered to ≥100% baseline eGFR, the threshold value at which a significant difference in 3-year MAE was observed was < 70% of baseline eGFR (Fig. 1c).

The Cox proportional hazards regression model showed that age, BMI, chronic heart failure, diabetes, severity of AKI and RRT were all risk factors for 3-year MAEs **(**Table 5**)**. After adjusting for the confounding factors, age, NYHA grading, diabetes and RRT according to ATN and ADQI criteria, AKI with complete renal recovery was still a risk factor for 3-year MAE (OR: 1.69, 95% CI: 1.20–2.38, *P* <  0.05; OR: 1.45, 95% CI: 1.03–2.04, *P* <  0.05). According to Pannu, KDIGO and Bucaloiu criteria, AKI with complete renal recovery was not a risk factor for 3-year MAE and using 5 different criteria, AKI with partial renal recovery was always a risk factor for 3-year MAE **(**Table 6**)**. We found that relative to the patients who recovered to within 0% of the baseline SCr, the threshold value at which a significant difference in 3-year MAE was observed was more than 30% above baseline SCr (Fig. 1a) or more than 0.4 mg/dL above baseline SCr (Fig. 1B). Relative to the patients who recovered to ≥100% baseline eGFR, the threshold value at which a significant difference in 3-year MAE was observed was < 70% of baseline eGFR (Fig. 1c).

**Table 5:** Cox regression model for risk factors of 3-year MAEs

	OR	95% CI	*P*-value
Age (every one year added)	1.058	1.043–1.074	< 0.001
BMI (every one unit added)	0.951	0.913–0.990	0.015
NYHA III-IV (Y/N)	1.786	1.291–2.470	< 0.001
Diabetes (Y/N)	3.741	2.761–506	< 0.001
no AKI (reference)			
AKI 1	1.537	1.080–2.188	0.017
AKI 2	3.493	2.304–5.298	< 0.001
AKI 3	4.295	2.762–6.680	< 0.001
RRT (Y/N)	6.823	4.223–11.026	< 0.001

*AKI*: Acute kidney injury; *BMI*: Body mass index; *MAE*: Major adverse events; *NYHA*: New York heart association; *RRT*: Renal replacement therapy

**Table 6:** Cox regression model for risk factors of 3-year MAEs under five complete renal recovery definitions adjusted for the confounding factors age, NYHA grading, diabetes and RRT

	ATN	ADQI	Pannu	KDIGO	Bucaloiu
Age (every one year added)	1.046 (1.033–1.060)	1.050 (1.037–1.063)	1.050 (1.036–1.064)	1.043 (1.030–1.057)	1.052 (1.038–1.066)
NYHA III-IV (Y/N)	1.437 (1.055–1.957)	1.557 (1.147–2.115)	1.675 (1.234–2.275)	1.641 (1.206–2.232)	1.626 (1.198–2.208)
Diabetes (Y/N)	4.240 (3.175–5.662)	4.043 (3.039–5.379)	3.890 (2.923–5.176)	4.338 (3.252–5.788)	4.035 (3.035–5.364)
RRT (Y/N)	5.363 (3.487–8.248)	6.145 (4.009–9.419)	9.555 (6.317–14.453)	9.446 (6.226–14.33)	11.018 (7.314–15.599)
no AKI (reference)					
AKI with complete recovery	1.69 (1.20–2.38)^*^	1.45 (1.03–2.04)^*^	1.17 (0.79–1.74)	1.37 (0.95–1.99)	1.37 (0.91–2.08)
AKI with partial recovery	15.25 (10.68–21.78)^*^	11.39 (8.09–16.02)^*^	4.82 (3.51–6.62)^*^	5.60 (4.01–7.81)^*^	3.62 (2.64–4.95)^*^

^*^P < 0.05

*ADQI*: Acute Dialysis Quality Initiative; *AKI*: acute kidney injury; *ATN*: Acute Renal Failure Trial Network; *BMI*: Body mass index; *KDIGO*: Kidney Disease: Improving Global Outcomes; *MAE*: major adverse events; *NYHA*: New York heart association; *RRT*: Renal replacement therapy

## Discussion

Kellum et.al noted that the development a good definition of renal recovery from AKI should include four main domains: inception (starting point for recovery); magnitude (threshold for recovery); timing (when recovery is assessed), and confounding factors (competing endpoints and interference) [15].

We found that using the five definitions of renal recovery (ATN, ADQI, Pannu, KDIGO, Bucaloiu), complete renal recovery was mostly derived from mild AKI patients, which accounted for more than 80%, while the majority of patients in the severe AKI group had partial renal recovery. These results involve the first key issue: inception of recovery, because recovery from mild AKI is quite different from severe AKI. The fact is that an increase of SCr in mild AKI can easily reach “complete recovery” after small decreases. When a severe AKI occurred, it is difficult to reach “complete recovery”. Moreover, we found that under five different definitions of complete renal recovery, SCr at discharge in the preoperative eGFR ≥90 mL/min/1.73 m^2^ group was significantly lower and eGFR at discharge was significantly higher than in the preoperative eGFR < 90 mL/min/1.73 m^2^ group. So we believe that inception for recovery is not only related to the extent of AKI but is also related to baseline renal function. Until now, all the definitions of renal recovery, except KDIGO criteria, used baseline renal function as a reference. It seems unreasonable that patients with renal dysfunction who recovered from AKI to the former “abnormal” level can be considered as “complete recovery” while patients with normal renal function had better recovery but were not within baseline SCr or eGFR i.e. “incomplete recovery”. The definition of AKI was different for acute and chronic diseases, so it may be more appropriate to make the definition for renal recovery according to the acute and chronic statuses.

In the present study, according to ATN and ADQI criteria, AKI with complete renal recovery was still a risk factor while according to Pannu, KDIGO and Bucaloiu criteria, AKI with complete renal recovery was not a risk factor for 3-year MAE. Our previous study reported that AKI with complete recovery was still a risk factor for long-term death or progressive CKD [1]. Proliferation of tubular cells may prompt recovery of SCr or eGFR after AKI, but glomerular hyperfiltration, mitochondrial dysregulation, endothelial injury, reduced capillary density and tubulo-interstitial inflammation/fibrosis may be the cause of long-term persistent injury [18]. Bucaloiu et al. defined “complete recovery” of AKI as eGFR within at least 90% of the baseline eGFR and a follow-up of 3.3 years. The results showed that any stage of AKI was associated with the development of CKD (adjusted HR 1.91, 95% CI: 1.75–2.09) [8]. Jones et al. defined “complete recovery” of AKI as SCr within > 10% of baseline and a follow-up for 2.5 years. The results revealed that the incidence of CKD in AKI patients was significantly higher than in non-AKI patients (15% vs 3%, *P* <  0.05, HR 5.93, 95% CI 4.49–7.84) [19]. Except in the above mentioned studies, most used criteria for “complete recovery” as a SCr return to within > 50% of baseline (ADQI). This threshold range is so wide that it leads to the result that AKI with complete recovery was still a risk factor for long-term death or CKD, which obviously diminished the importance of “complete recovery”. Our study confirmed that if the threshold criteria can be narrower than KDIGO, Pannu or Bucaloiu, AKI with complete recovery may not be a risk factor for long-term death or CKD and only AKI with partial renal recovery will always be a risk factor. Thus, complete recovery from AKI is very important, which can diminish the relationship between AKI and ESRD.

We tried to find the thresholds for significant renal recovery in relation to 3-year MAEs. The results showed that the cut-off point at which a significant difference in 3-year MAEs was observed was when non-recovery was defined as > 30% and > 0.4 mg/dL above baseline SCr, or < 70% of baseline eGFR (Fig. 1). For a long time, the most commonly used fixed threshold for “complete recovery” was eGFR at discharge ≥60 mL/min/1.73 m^2^ (KDIGO criteria), because anything below this level is defined as CKD [6]. Considering that it was not suitable for patients with previous renal dysfunction, ADQI recommended a recovery threshold of 50% above baseline for “complete recovery” [7], but Pannu et al. found that mortality differences became significantly different between groups when recovery was defined as within 55% of the baseline. However, a value within 25% of baseline was associated with an increased risk if the endpoint was ESRD [9], a result similar to our findings.

Timing is also a critical factor in recovery, which can determine if it is a “true” recovery. Timing can refer to how persistent an episode of AKI is or how sustained the recovery is or when recovery is assessed. Chawla et al. defined five different recovery patterns from AKI and found the most common was early reversal that was sustained throughout discharge (26.6%), followed by late reversal after day 7 (9.7%), early reversal with one or more relapses but with ultimate recovery (22.5%) and relapse without recovery (14.7%). Relapses are associated with a 5-fold increased risk of death at 1 year compared to early sustained reversal [20].

The Cox regression model used in our study showed that age, BMI, diabetes, New York heart association (NYHA) > II, severity of AKI and RRT were all independent risk factors for 3-year MAEs. Chawla et al. designed prediction models for progression to stage 4 CKD, which included age, severity of AKI, time at risk and baseline serum albumin concentrations, and the results showed good predictive accuracy (‘c’ 0.81–0.82) in a model validation [21]. However, the progression from AKI to CKD was “dose” related. The long-term outcome will become worse when the AKI stage is more severe or the episode of AKI is longer or the kidney received more insults, which should be considered in prediction models. As a retrospective study, we acknowledge the limitations of our study. It was impossible for us to record the persistence of AKI episodes or if the recovery was sustained, an issue which requires further long term studies.

## Conclusions

ADQI or ATN-equivalent criteria may overestimate the extent of renal recovery of cardiac surgery associated AKI patients. KDIGO, Pannu and Bucaloiu equivalent criteria may be more appropriate for clinical use. According to our results, SCr at discharge ≤30% above baseline SCr or ≤ 0.4 mg/dL above baseline SCr, and eGFR at discharge ≥70% of baseline eGFR can be considered as “complete recovery”, which may serve as a new definition for renal recovery in the future.

## Data Availability

The datasets used and/or analyzed during the current study are available from the corresponding author on reasonable request.

## Abbreviations

ADQI: Acute Dialysis Quality Initiative
AKI: Acute kidney injury
ATN: Acute Renal Failure Trial Network
BMI: Body mass index
BUN: Blood urea nitrogen
CABG: Coronary artery bypass graftin
CKD: Chronic kidney disease
CKD-EPI: Chronic Kidney Disease Epidemiology Collaboration equation
CSA-AKI: Cardiac surgery associated acute kidney injury
eGFR: Estimated glomerular filtration rate
ESRD: End stage renal disease
GDRRT: Goal-directed renal replacement therapy
ICU: Intensive care unit
KDIGO: Kidney Disease: Improving Global Outcomes
MAE: Major adverse events
NYHA: New York heart association
RRT: Renal replacement therapy
SCr: Serum creatinine

## References

[1] Xu JR, Zhu JM, Jiang J, Ding XQ, Fang Y, Shen B (2015). Risk factors for long-term mortality and progressive chronic Kidney disease associated with acute Kidney injury after cardiac surgery. Medicine.

[2] Jiang W, Teng J, Xu J, Shen B, Wang Y, Fang Y, et al. Dynamic Predictive Scores for Cardiac Surgery-Associated Acute Kidney Injury. J Am Heart Assoc. 2016;5(8). 10.1161/JAHA.116.003754 PMC5015294 27491837

[3] Xu J, Shen B, Fang Y, Liu Z, Zou J, Liu L (2015). Postoperative fluid overload is a useful predictor of the short-term outcome of renal replacement therapy for acute Kidney injury after cardiac surgery. Medicine.

[4] Li P, Qu LP, Qi D, Shen B, Wang YM, Xu JR (2017). Significance of perioperative goal-directed hemodynamic approach in preventing postoperative complications in patients after cardiac surgery: a meta-analysis and systematic review. Ann Med.

[5] Xu J, Ding X, Fang Y, Shen B, Liu Z, Zou J (2014). New, goal-directed approach to renal replacement therapy improves acute kidney injury treatment after cardiac surgery. J Cardiothorac Surg.

[6] Kidney Disease Improving Gloabl Outcomes (KDIGO) Acute Kidney Injury Work Group. KDIGO Clinical Practice Guideline for Acute kidney Injury. 2012.

[7] Bellomo R, Ronco C, Kellum JA, Mehta RL, Palevsky P (2004). Acute Dialysis quality initiative w. acute renal failure - definition, outcome measures, animal models, fluid therapy and information technology needs: the second international consensus conference of the acute Dialysis quality initiative (ADQI) group. Crit Care.

[8] Bucaloiu ID, Kirchner HL, Norfolk ER, Hartle JE, Perkins RM (2012). Increased risk of death and de novo chronic kidney disease following reversible acute kidney injury. Kidney Int.

[9] Pannu N, James M, Hemmelgarn B, Klarenbach S (2013). Alberta Kidney disease N. association between AKI, recovery of renal function, and long-term outcomes after hospital discharge. Clin J Am Soc Nephrol.

[10] Palevsky PM, Zhang JH, O'Connor TZ, Chertow GM, Crowley ST, Choudhury D (2008). Intensity of renal support in critically ill patients with acute kidney injury. N Engl J Med.

[11] Doyle JF, Forni LG (2015). Long-term follow-up of acute Kidney injury. Crit Care Clin.

[12] Harel Z, Wald R, Bargman JM, Mamdani M, Etchells E, Garg AX (2013). Nephrologist follow-up improves all-cause mortality of severe acute kidney injury survivors. Kidney Int.

[13] Smart NA, Dieberg G, Ladhani M, Titus T (2014). Early referral to specialist nephrology services for preventing the progression to end-stage kidney disease. Cochrane Database Syst Rev.

[14] Kirwan CJ, Blunden MJ, Dobbie H, James A, Nedungadi A, Prowle JR (2015). Critically ill patients requiring acute renal replacement therapy are at an increased risk of long-term renal dysfunction, but rarely receive specialist nephrology follow-up. Nephron.

[15] Kellum JA (2014). How can we define recovery after acute kidney injury? Considerations from epidemiology and clinical trial design. Nephron Clin Pract.

[16] National Kidney, Foundation. K/DOQI clinical practice guidelines for chronic kidney disease: evaluation, classification, and stratification. American journal of kidney diseases : the official journal of the National Kidney Foundation 2002;39(2 Suppl 1):S1–266. 11904577

[17] Shaw A (2011). Models of preventable disease: contrast-induced nephropathy and cardiac surgery-associated acute kidney injury. Contrib Nephrol.

[18] Varrier M, Forni LG, Ostermann M (2015). Long-term sequelae from acute kidney injury: potential mechanisms for the observed poor renal outcomes. Crit Care.

[19] Jones J, Holmen J, De Graauw J, Jovanovich A, Thornton S, Chonchol M (2012). Association of complete recovery from acute kidney injury with incident CKD stage 3 and all-cause mortality. Am J Kidney Dis.

[20] Kellum JA, Sileanu FE, Bihorac A, Hoste EA, Chawla LS (2017). Recovery after acute Kidney injury. Am J Respir Crit Care Med.

[21] Chawla LS, Amdur RL, Amodeo S, Kimmel PL, Palant CE (2011). The severity of acute kidney injury predicts progression to chronic kidney disease. Kidney Int.

